# Prevalence of Overweight in Children Starts Early in Life: Findings From the Italian NASCITA Birth Cohort Study

**DOI:** 10.1155/jobe/1633187

**Published:** 2026-04-19

**Authors:** Antonio Clavenna, Rita Campi, Chiara Pandolfini, Maurizio Bonati

**Affiliations:** ^1^ Laboratory of Child Health and Development Epidemiology, Istituto di Ricerche Farmacologiche Mario Negri IRCCS, Milan, Italy, marionegri.it

**Keywords:** birth cohort, children, growth, overweight

## Abstract

**Objective:**

To estimate the prevalence of overweight at 36 months of age and that of persistent overweight in an Italian birth cohort and to identify factors related to an increased likelihood of having overweight.

**Methods:**

The Italian NASCITA birth cohort was analysed. Children were classified in the underweight, normal or overweight range at 12, 24 and 36 months of age according to the World Health Organization percentiles, and the prevalence of overweight (≥ 85th centile) was estimated. Persistent overweight was defined as having overweight in all three assessments. To test the association between the chance of having overweight, and parental and child characteristics, healthy newborns with appropriate for gestational age birth weight were selected, and univariate and multivariate analyses were performed.

**Results:**

The prevalence of overweight was 22.7% at 12 months of age and 21.2% at 36 months (chi square‐for‐trend = 1.5 and *p* = 0.21). In all, 8.8% of the children had persistent overweight. Overweight at 12 months (RR: 3.28 and 95% CI: 2.69–4.00) and a big appetite (RR: 2.00 and 95% CI: 1.59–2.52) were the main factors associated with greater likelihood of overweight at 36 months, while excessive appetite and frequency of interaction with electronic devices were the main determinants of persistent overweight.

**Conclusions:**

The body mass index status at 12 months greatly influenced that at 36 months. The increased risk of persistent overweight in children interacting with electronic devices suggests that extreme caution in allowing preschool children to use smartphone or tablets should be adopted. Furthermore, nutritional education of the entire family is essential to appropriately guide children’s appetite.

## 1. Introduction

Childhood overweight and obesity represent an increasing public health issue that threatens future health and quality of life [1, 2]. It is estimated that nearly 39 million children less than 5 years old are living with overweight or obesity globally [2], and preschoolers with these conditions often have overweight also in adolescence or adulthood [3–5]. Obesity prevention should, therefore, start in early childhood [6].

The development of obesity may be influenced by early‐life factors (e.g., maternal body mass index [BMI], gestational weight gain, maternal smoking habits and infant feeding) [7, 8]. Among these, an important role in growth and development is played by nutrition, with breastfeeding recommended by the World Health Organization (WHO) as the best method to nourish infants due to its positive short and long‐term health effects on children and mothers [9]. Several studies found an association between breastfeeding and a reduced risk of being overweight, even if results are not conclusive [10–12]. Another factor associated with an increased risk of being overweight is the length of time spent in front of screens due to use of electronic devices, given the related sedentary behaviour [13–15].

Italy is one of the European countries with the greatest prevalence of childhood overweight and obesity, with a rate in 8‐year‐old children of 42% and 21%, respectively [16, 17]. A North–South gradient has been observed, and school‐aged children living in southern Italy have a 2‐fold greater risk of overweight compared with children living in the North [18]. There is, however, scant information concerning the proportion of overweight Italian children at an early age.

In this context, data from the Italian NASCITA (NAscere e creSCere in ITAlia) birth cohort study were recently used to assess the prevalence of infants at risk at 12 months of age, and an estimate of 23.1% was found [19]. Additionally, the variables more strongly associated with a greater likelihood of overweight were found to be excessive infant appetite reported by parents, living in southern Italy and traditional weaning [19].

The authors then wished to evaluate the prevalence of overweight at different ages, the percentage of children with persistent overweight and the factors associated with BMI status. A follow‐up was, therefore, performed at 36 months of age and is described in this article.

## 2. Methods

### 2.1. Data Source

The NASCITA birth cohort was set up by the Laboratory for Mother and Child Health of the Istituto di Ricerche Farmacologiche Mario Negri IRCCS in Milan in collaboration with the national Paediatric Cultural Association (ACP).

**FIGURE 1:**
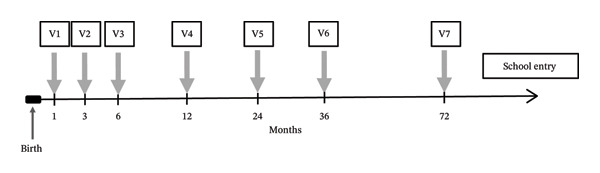
Timeline of well‐child visits in the NASCITA study.

The methods of the NASCITA cohort have been described elsewhere [19–21]. Briefly, all Italian children receive primary healthcare exclusively from a family paediatrician until they are at least 6 years old as part of universalistic health system organization. Seven well‐child visits are scheduled by the paediatrician in the first 6 years of a child’s life to monitor growth and development and offer preventive care (Figure 1) [20]. Additional visits are guaranteed when needed. The newborn population consists of all infants born during the enrolment period (1 April 2019–31 July 2020) and seen by the paediatricians for the well‐child visits of the first year of life, if parental consent was given [21].

Some information was obtained for the NASCITA study in addition to the data routinely collected by the paediatricians during the well‐child visits [19–21].

### 2.2. Outcomes

Family paediatricians measured the height and weight of children at 12, 24 and 36 months of age during well‐child visits using a standardised protocol developed by Italian paediatric scientific societies [22]. BMI was then calculated using these measurements.

Infants were classified as underweight (< 5th centile), normal (5th–84th centile) and overweight (≥ 85th centile) at visits 4, 5 and 6 according to the WHO percentiles of BMI, estimated on the basis of the gender of the neonate and the age at the moment of the visit [23].

The likelihood of being overweight at 36 months of age was the primary outcome measure. Moreover, the prevalence at 12 and 24 months and the prevalence of overweight at all three timepoints (persistent overweight) were estimated.

Age‐ and sex‐specific 36‐month BMI z‐scores were calculated using the WHO BMI reference data [23].

### 2.3. Healthy AGA Subsample

Consistently with the previous study, for the evaluation of factors associated with an increased BMI, healthy newborns were selected (i.e., with the exclusion of preterm and low birth weight newborns and of neonates with congenital malformations and/or admitted to an intensive care unit) [19]. Moreover, the analyses were focused on neonates with appropriate for gestational age (AGA) birth weight, estimated using the Italian Neonatal Study (INeS) charts [24], in order to monitor a cohort with homogeneous characteristics and a similar baseline risk, consistently with most of the studies.

### 2.4. Covariates

Variables associated with an increased risk of overweight in childhood in previous studies were selected as covariates [7, 8, 19].

The full list of covariates is reported in the Supporting information.

### 2.5. Statistical Analysis

Chi‐square tests were performed with the aim to evaluate the association between the reported covariates and the BMI status (overweight versus normal; persistent overweight: yes versus no), and a Poisson regression model with robust variance was performed. Covariates were considered eligible for inclusion in the multivariate model if the corresponding *p* value in the univariate analysis was ≤ 0.2.

Multiple linear regression models were used to examine the associations between covariates and 36‐month BMI z‐score.

Statistical significance was evaluated using a two‐tailed *p* value < 0.05. All data management and analyses were performed using SAS software.

### 2.6. Ethical Statements

The study was approved by the Fondazione IRCCS Istituto Neurologico “Carlo Besta” ethics committee (Verbale n 59, 6th February 2019), and informed consent was obtained from the newborns’ parents.

## 3. Results

It was possible to collect data at 36 months of age for 1734 out 2835 (61.1%) enrolled infants with the BMI evaluation at 12 months. A total of 1101 children missed one or more well‐child visits, mainly due to the impact of the COVID‐19 pandemic (e.g., parental anxiety and difficulty accessing a paediatrician’s office). The sociodemographic characteristics of the families involved, as well as a comparison between assessable versus nonassessable families, are reported in Table S1. No differences were observed between the two samples, with the exception of the proportion of single mothers and of primiparous women (0.7% versus 3.6% and 52.8% versus 58.1%, respectively).

**FIGURE 2:**
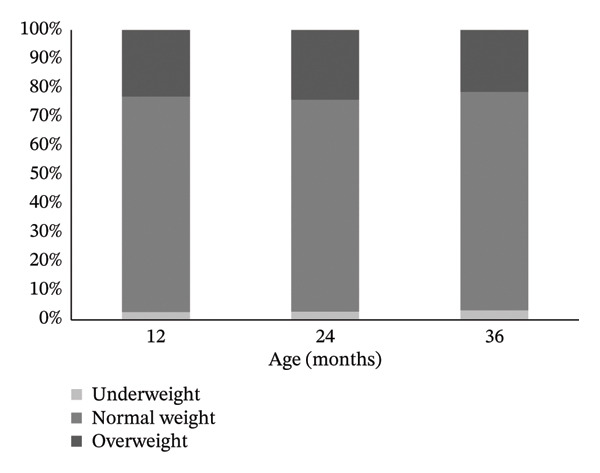
BMI status at 12, 24 and 36 months of age.

The prevalence of overweight was 22.7% (95% CI: 20.8%–24.7%) at 12 months of age, 23.9% (95% CI: 21.9%–26.0%) at 24 months and 21.2% (95% CI: 19.3–23.1) at 36 months ($\chi_{t}^{2}$ = 1.5 and *p* = 0.21) while that of underweight ranged from 2.7% to 3.2% (Figure 2).

### 3.1. Overweight at 36 Months

A few variables at 12 months of age were associated with different likelihoods of being overweight at 36 months of age at the univariate analyses (Table S2). More specifically, the variables more strongly associated, at the Poisson regression analysis (Table 1), were overweight at 12 months of age (RR: 3.28 and 95% CI: 2.69–4.00), an excessive appetite (RR: 2.00 and 95% CI: 1.59–2.52) and a mother with overweight before pregnancy (RR: 1.35 and 95% CI: 1.11–1.65).

**TABLE 1:** Variables associated with overweight at 36 months with the Poisson regression analysis.

Variable	Value	Risk ratio (95% CI)	*p* value
Setting	Rural	0.73 (0.61–0.88)	0.001
	Urban	1	

Prepregnancy BMI	Underweight	0.68 (0.40–1.15)	0.15
	Normal	1	
	Overweight	1.35 (1.11–1.65)	0.003

Infant appetite^∗^	Poor	0.41 (0.15–1.14)	0.09
	Medium	1	—
	Big	2.00 (1.59–2.52)	< 0.001

Overweight at 12 months	Yes	3.28 (2.69–4.00)	< 0.001
	No	1	

^∗^Infant appetite as perceived by the parents.

Table 2 reports the variables with an impact on BMI z score at 36 months of age. Most of the variables were the same identified in the Poisson regression analysis. The exceptions were maternal unemployment (*β* = 0.133) and early weaning (*β* = 0.168). Infant appetite had the greatest influence, with *β* = 0.880 for a big appetite and *β* = −0.625 for a poor appetite.

**TABLE 2:** Results of the multiple linear regression (dependent variable: BMI z score at visit 6).

Variable	Beta	SE	*p* value
Intercept	0.30	0.051	< 0.0001
Setting = rural	−0.153	0.057	0.007
Mother unemployed	0.133	0.0430	0.006
Prepregnancy BMI = underweight	−0.307	0.111	0.006
Prepregnancy BMI = overweight	0.215	0.063	0.0007
Timing of weaning < 5 months	0.168	0.077	0.0287
Infant appetite = Poor^∗^	−0.625	0.128	< 0.0001
Infant appetite = Big^∗^	0.880	0.089	< 0.0001
Parental concern about growth = yes	−0.309	0.083	0.0002

^∗^Infant appetite as perceived by the parents.

### 3.2. Persistent Overweight

In all, 195 out of 388 (50.3%) children with overweight at 12 months of age were in the overweight range at 36 months, 152 (8.8% of the overall sample) of which at all 3 visits.

The variables associated at the univariate analysis with a greater likelihood of having persistent overweight were living in the South of Italy, maternal BMI before pregnancy, type of weaning, child’s appetite and frequency of interaction with digital devices (Table S3). At the Poisson regression analysis, the variables were excessive child appetite (RR: 3.28 and 95% CI: 2.25–4.79; *p* < 0.0001), high (RR: 2.05 and 95% CI: 1.09–3.85; *p* = 0.03) or medium (RR: 2.03 and 95% CI: 1.18–3.52; *p* = 0.01) frequency of electronic device interaction and traditional weaning (RR: 1.75 and 95% CI: 1.09–2.81; *p* = 0.02).

## 4. Discussion

Different estimates of overweight in preschoolers were reported in previous international studies, with a range between 12% and 30% [25–28].

In our sample, the estimate was of more than one out of five children at 3 years of age, according to the WHO growth chart, with a prevalence that did not significantly change with the child’s age. From this point of view, it seems that Italian children are in the upper range for the prevalence of overweight.

In half of children with overweight at 12 months, this condition persisted in the subsequent years, with the BMI status at 12 months of age greatly influencing that at 36 months. This finding is consistent with data underlying the importance of early childhood in determining the status in childhood and adulthood [3–5].

Some variables that were strongly associated with overweight at 12 months [19] did not influence the BMI status at 3 years of age, for example, geographical area of residence and maternal unemployment, even if this latter was associated with an increase in the BMI z‐score.

On the contrary, the maternal BMI before pregnancy was associated with a greater likelihood of overweight at 36 months but not at 12 months.

In this regard, the findings of the 36‐month evaluation seem more consistent with the results of other studies, since prepregnancy overweight has previously been associated with a greater BMI in infancy and childhood [29–34]. Other results that confirm findings from previous studies were the fact that having a “big appetite” is a trait associated with a greater risk of overweight [35–37] and that there is an association between overweight and screen time [13–15]. On the contrary, in our sample, we did not confirm an association between full‐time employment of the mother and an increased chance of being overweight in children [38–41].

It is interesting to note that in our study, an excessive appetite resulted as the main variable associated with a greater likelihood of having a persistent overweight, while when evaluating the overweight at 36 months, the main determinant was having overweight at 12 months. Prepregnancy overweight and excessive child appetite are related to family eating habits and are part of a food culture that should be addressed with parents at every opportunity [42].

We observed an association between interaction with electronic devices and risk of persistent overweight, while this variable did not influence the likelihood of overweight at 36 months alone. However, this finding should be interpreted cautiously since the use of electronic devices may be a proxy for broader family routines, parenting practices or underlying sociodemographic characteristics.

Traditional weaning, when compared with BLW, was associated with a greater likelihood of overweight at 12 months [19]. Even if the association was not confirmed at 36 months of age, this variable increased the chance of having a persistent overweight.

The association between BLW and lower BMI was observed in previous studies [43, 44], and it has been hypothesized that BLW may improve the infant’s appetite control and lead to higher levels of satiety responsiveness [44].

In the previous evaluation of overweight at 12 months of age [19], the geographical area of residence resulted the main risk factor, but also, this finding was not confirmed at 36 months. Living in the South of Italy was associated with an increased likelihood of persistent overweight with the univariate analysis but not with the multivariate. Differences between North and South of Italy have been described in school‐aged children, and the influence of area of residence was therefore expected, but it is likely mediated by other variables, such as the infant’s appetite, the frequency of interaction with electronic devices or the type of weaning [18, 45].

### 4.1. Strengths and Limitations

The sample was representative of the national population for distribution by geographical area of residence, environment (rural/urban), and demographic characteristics of the families [21]. The anthropometric measures were collected by the paediatricians during the visits and, from this point of view, may be more accurate than when recorded by parents.

Some limitations should be acknowledged.

Study design: Due to the observational nature of the study, the analysis can only identify variables associated with a higher likelihood of overweight and does not allow for causal inferences to be made. Furthermore, the NASCITA study was not specifically designed to investigate the determinants of being overweight. The present analyses, therefore, relied on data collected during routine well‐child visits, supplemented with additional information.

Sampling and generalizability: The family paediatricians participated on a voluntary basis and most of them were aware of the best practices for supporting early child development. It is possible that these paediatricians are not fully representative of Italian paediatricians in general, and, in particular, may be more sensitive to infant feeding and nutrition.

The COVID‐19 pandemic and the subsequent epidemics of bronchiolitis, flu and scarlet fever represented a burden for the paediatricians’ activities. In some cases well‐child visits were postponed, and some participants faced difficulties in continuing the data collection for the study. Nevertheless, no significant differences were observed between the sociodemographic characteristics of children who completed all follow‐up visits and those who did not.

In addition, in the evaluation of factors associated with a greater likelihood of overweight, we choose to include, consistently with other studies, only healthy newborns with an AGA birth weight. This was done to monitor a cohort with the same baseline risk, and our results may apply only to these neonates. In any case, when considering all healthy newborns independently of their weight for gestational age, the prevalence of overweight was very close to that observed in AGA (at 36 months, 21.2% versus 21.3%).

#### 4.1.1. Measurement Issues

The validity of BMI as a predictor of adiposity and in the body composition assessment is a matter of debate [46], yet it is a widely used measure, even in the preschool population [25–28, 47]. Moreover, the WHO growth standards were used to define overweight, in line with the Italian scientific societies’ recommendations for diagnosing paediatric obesity [48]. While the prevalence of overweight may differ when using other growth curves [49, 50], such differences are expected to be minimal for children aged 2‐3.

We were not able to collect information on the children’s dietary intake and the quality of their diets. Our definition of baby‐led weaning was broad and included different attitudes. We did not, for example, have data concerning the percentage of children receiving spoon feeding or puree feeding. The estimate of screen time and frequency of interaction was reported by parents during the well‐child visits using broad categories, and it was not possible to collect data about the actual mean duration of using screen/electronic devices [13]. Despite this limitation, a frequent interaction with electronic devices was one of the factors associated with persistent overweight.

## 5. Conclusion

More than one out of five infants in Italy have overweight, and in nearly one out of ten, the overweight is persistent. The BMI status at 12 months of age influenced the likelihood of having overweight in the following years, and the frequency of interactions with electronic devices increased the likelihood of persistency of overweight, whereas baby‐led weaning seemed to have a protective role. Finally, educating the mother about nutrition, even before pregnancy, as well as educating the entire family, should play a key role in appropriately guiding children’s appetite.

## Author Contributions

Antonio Clavenna, Maurizio Bonati, Chiara Pandolfini and Rita Campi conceived and designed the study. Rita Campi analysed the data. Antonio Clavenna wrote the first draft of the manuscript. Rita Campi, Chiara Pandolfini and Maurizio Bonati provided critical revisions.

## Funding

This work was partly supported by an economic contribution by the Associazione Amici del Mario Negri.

## Disclosure

The Associazione Amici del Mario Negri had no role in the design and conduct of the study. All authors have read and approved the submitted manuscript.

## Conflicts of Interest

The authors declare no conflicts of interest.

## Supporting Information

Supporting methods section reports the list and the definition of the covariates.

Table S1: The supporting Table 1 (S1) reports a comparison of sociodemographic characteristics between families of children included in analyses (*n* = 1734) and families of children who were not assessable due to missing visits at 24 and/or 36 months (*n* = 1101).

Table S2: The supporting Table 2 (S2) reports the results of the univariate analysis evaluating the association between maternal and neonatal characteristics and BMI at 36 months (overweight versus normal).

Table S3: The supporting Table 3 (S3) reports the results of the univariate analysis evaluating the association between maternal and neonatal characteristics and persistent overweight.

## Supporting information


**Supporting Information** Additional supporting information can be found online in the Supporting Information section.

## Data Availability

The data that support the findings of this study are available from the corresponding author upon reasonable request.
