# Supplementary material for: Prevalence of Overweight in Children Starts Early in Life: Findings From the Italian NASCITA Birth Cohort Study
Source: J Obes. 2026 Apr 19;2026:1633187. doi: 10.1155/jobe/1633187 (PMC13092800; doi:10.1155/jobe/1633187)
Supplement: Supplementary file 1 — Supporting Information Additional supporting information can be found online in the Supporting Information section. [file JOBE-2026-1633187-s001.docx]

**Prevalence of overweight in children starts early in life: findings from the Italian NASCITA birth cohort study**

Antonio Clavenna, Rita Campi, Chiara Pandolfini, Maurizio Bonati

**Affiliation:** Laboratory of Child Health and Development Epidemiology, Istituto di Ricerche Farmacologiche Mario Negri IRCCS, Milan, Italy

**Corresponding author**: Antonio Clavenna, Laboratory of Child Health and Development Epidemiology, Istituto di Ricerche Farmacologiche Mario Negri IRCCS, via Mario Negri 2, Milan 20156 Italy. E-mail: antonio.clavenna@marionegri.it

**SUPPLEMENTARY METHODS SECTION**

**List of covariates**

Variables associated with an increased risk of overweight in childhood in previous studies were selected as covariates [1–3]. They include:

*- maternal characteristics:*

geographical area of residence (North/Center/South), age of the mother at delivery, maternal educational level (low: no schooling or primary school; high: secondary school or university),

employment status, marital status, nation of birth (Italy yes/no), and parity (primiparous yes/no).

*- data concerning pregnancy, delivery and newborn:*

pre-pregnancy BMI, gestational weight gain, occurrence of gestational diabetes, type of delivery, gender of the neonate.

Mothers were grouped according to their pre-pregnancy BMI into three categories, underweight (≤18.5), normal (18.6-24.9), and overweight or obese (≥25.0). To evaluate gestational weight gain, the weight variations recommended by the Institute of Medicine criteria were applied after grouping the mothers according to the pre-pregnancy BMI [4,5].

*- lifestyle habits:*

smoking habits of the mother (mother smoked during pregnancy and or postpartum period), mean number of daily hours spent outdoors by parents and infant (<1: score=1; 1-3: score=2; >3: score=3), frequency of screen exposure in watching videos (Never=1, Sometimes=2, Daily =3), and direct interaction with electronic devices (Never=1, Sometimes=2, Daily/Nearly daily=3). The time spent for outdoor activities, the frequency of screen exposure, and interaction with electronic devices were evaluated at 12, 24 and 36 months of age, and the score obtained at each visit was summed to obtain an overall score. For these three variables the overall frequency was categorized in: low - total score≤4; medium - total score=5-6; high - total score: ≥7.

*- infant nutrition:*

breastfeeding practice (exclusive breastfeeding ≥6 months, EBF; breastfeeding ≥12 months/≥24 months), timing of weaning according to the age of the infant in months (<5; 5; ≥6), type of weaning (traditional versus baby-led, BLW), consumption of commercial baby food (yes/no), parental perception of general infant appetite (poor/normal/big), and parental concern about growth (yes/no).

Traditional weaning involved spoon feeding purees or semi-liquid foods then graduating to more textured foods and some finger foods before joining in with the family diet, while BLW included different approaches (e.g., “on demand approach/self-weaning”, low degree of spoon feeding or puree feeding) [6].

The perception of parents of their infant’s appetite was investigated by the pediatricians by adapting the correspondent item in the Baby Eating Behavior Questionnaire [7].

Parental perception of infant appetite was evaluated at 12, 24 and 36 months. For each visit, a score was given: poor=1, normal=2, excessive=3. The overall assessment was ≤4=poor; 5-6=normal; ≥7=excessive.

*- occurrence of sleep disorders*

If parents reported a sleep disorders (mainly recurrent night awakenings) in at least one of the visits, the variable was “Yes”, otherwise it was “No”.

With the exception of the anthropometric measures, all the other data were collected by the family pediatricians through an interview with the parents. The details concerning data collection were reported in the study protocol [8]. Information on maternal characteristics, and on pregnancy, delivery, and neonates were collected during visit 1, while those on lifestyle habits and infant nutrition during visits 1-6.

**References**

1 Woo Baidal JA, Locks LM, Cheng ER, *et al.* Risk Factors for Childhood Obesity in the First 1,000 Days: A Systematic Review. *Am J Prev Med*. 2016;50:761–79. doi: 10.1016/j.amepre.2015.11.012

2 Monasta L, Batty GD, Cattaneo A, *et al.* Early-life determinants of overweight and obesity: a review of systematic reviews. *Obes Rev*. 2010;11:695–708. doi: 10.1111/j.1467-789X.2010.00735.x

3 Clavenna A, Morabito E, Cartabia M, *et al.* National, longitudinal NASCITA birth cohort study: prevalence of overweight at 12 months of age in children born healthy. *BMJ Paediatr Open*. 2023;7:e001622. doi: 10.1136/bmjpo-2022-001622

4 Nucci D, Chiavarini M, Duca E, *et al.* Pre-pregnancy body mass index, gestational weight gain and adverse birth outcomes: some evidence from Italy. *Ann Ig*. 2018;30:140–52. doi: 10.7416/ai.2018.2205

5 Benvenuti MB, Bø K, Draghi S, *et al.* The weight of motherhood: Identifying obesity, gestational weight gain and physical activity level of Italian pregnant women. *Womens Health (Lond)*. 2021;17:17455065211016136. doi: 10.1177/17455065211016136

6 Addessi E, Galloway AT, Wingrove T, *et al.* Baby-led weaning in Italy and potential implications for infant development. *Appetite*. 2021;164:105286. doi: 10.1016/j.appet.2021.105286

7 Llewellyn CH, van Jaarsveld CHM, Johnson L, *et al.* Development and factor structure of the Baby Eating Behaviour Questionnaire in the Gemini birth cohort. *Appetite*. 2011;57:388–96. doi: 10.1016/j.appet.2011.05.324

8 Pansieri C, Clavenna A, Pandolfini C, *et al.* NASCITA Italian birth cohort study: a study protocol. *BMC Pediatr*. 2020;20:80. doi: 10.1186/s12887-020-1961-1

**Supplementary Table 1 – Socio-demographic characteristics of the families involved, comparing the population of children included in the analyses (N= 1,734)** **with those not assessable due to missing visits at 24 and/or 36 months of age (n = 1,101).**

| **Variable** | ***Value*** | **Follow-up at 36 months** | | **p-value** |
| --- | --- | --- | --- | --- |
|  |  | Yes  (N=1734) | No  (N=1101) |  |
| Geographical area of residence | *North* | 856 (49.4) | 516 (46.9) | 0.43 |
|  | *Center* | 324 (18.7) | 214 (19.4) |  |
|  | *South* | 554 (31.9) | 371 (33.7) |  |
| Setting | *Urban* | 643 (37.1) | 447 (40.6) | 0.06 |
|  | *Rural* | 1091 (62.9) | 653 (59.4) |  |
| Maternal age at delivery | *Median (Interquartile range)* | 33.0 (30.0-37.0) | 33.0 (29.0-36.0) | 0.10 |
|  | *Mean (SD)* | 33.1 (5.2) | 32.7 (5.4) |  |
| Maternal educational level* | *High* | 1474 (85.7) | 938 (86.1) | 0.77 |
|  | *Low* | 245 (14.3) | 151 (13.9) |  |
| Maternal employment status | *Employed* | 1329 (71.8) | 771 (70.6) | 0.48 |
|  | *Unemployed* | 486 (28.2) | 321 (29.4) |  |
| Mother born in Italy | *Yes* | 1531 (88.4) | 955 (287.1) | 0.32 |
|  | *No* | 201 (11.6) | 141 (12.9) |  |
| Marital status | *With partner* | 1721 (99.3) | 1047 (21.1) | <0.0001 |
|  | *Single* | 13 (0.7) | 39 (3.6) |  |
| Primiparous | *Yes* | 911 (52.8) | 638 (58.1) | 0.007 |
|  | *No* | 813 (47.2) | 461 (41.9) |  |
| Type of delivery | *Spontaneous* | 1259 (72.6) | 789 (71.7) | 0.61 |
|  | *Caesarean* | 475 (27.4) | 311 (28.3) |  |
| Newborn gender | *Male* | 871 (50.2) | 551 (50.0) | 0.92 |
|  | *Female* | 863 (49.8) | 550 (50.0) |  |

***** Educational level: low: no schooling or primary versus high: secondary school or university

**Supplementary table 2 - Association between maternal and neonatal characteristics and BMI at 36 months (overweight versus normal).**

| **Variable** | ***Value*** | **BMI at 36 months** | | **OR (95%CI)** | **p-value** |
| --- | --- | --- | --- | --- | --- |
|  |  | Normal  (N=1343) | Overweight  (N=360) |  |  |
| Geographical area of residence | *North* | 675 (79.9) | 170 (20.1) | 1 | 0.45 |
|  | *Center* | 241 (76.5) | 74 (23.5) | 1.22 (0.89-1.66) |  |
|  | *South* | 427 (78.6) | 116 (21.4) | 1.08 (0.83-1.41) |  |
| Setting | *Urban* | 477 (76.2) | 149 (23.8) | 1 | 0.04 |
|  | *Rural* | 866 (80.4) | 211 (19.6) | 0.78 (0.62-0.99) |  |
| Maternal age at delivery | *Median (Interquartile range)* | 33.0 (30.0-36.0) | 32.0 (29.0-36.5) | Not applicable | 0.29 |
|  | *Mean (SD)* | 33.2 (5.1) | 32.9 (5.6) |  |  |
| Educational level** | *High* | 1144 (79.0) | 304 (21.0) | 1 | 0.70 |
|  | *Low* | 187 (77.9) | 53 (22.1) | 1.07 (0.77-1.49) |  |
| Employment status | *Employed* | 971 (80.0) | 243 (20.0) | 1 | 0.09 |
|  | *Unemployed* | 366 (76.2) | 114 (23.8) | 1.24 (0.97-1.98) |  |
| Mother born in Italy | *Yes* | 1191 (79.2) | 312 (20.8) | 1 | 0.42 |
|  | *No* | 152 (76.8) | 46 (23.2) | 1.16 (0.81-1.64) |  |
| Marital status | *With partner* | 1334 (78.9) | 357 (21.1) | 1 | 0.73 |
|  | *Single* | 9 (75.0) | 3 (25.0) | 1.25 (0.34-4.62) |  |
| Primiparous | *Yes* | 712 (79.8) | 180 (20.2) | 0.90 (0.72-1.14) | 0.40 |
|  | *No* | 626 (78.2) | 175 (21.8) | 1 |  |
| Pre-pregnancy BMI | *Underweight* | 98 (89.9) | 11 (10.1) | 0.45 (0.24-0.86) | <0.001 |
|  | *Normal* | 919 (80.1) | 228 (19.9) | 1 |  |
|  | *Overweight* | 297 (72.1) | 115 (27.9) | 1.56 (1.20-2.02) |  |
| Gestational weight gain | *Below* | 465 (81.2) | 108 (18.8) | 0.85 (0.64-1.12) | 0.17 |
|  | *Normal* | 513 (78.4) | 141 (21.6) | 1 |  |
|  | *Over* | 328 (76.3) | 102 (23.7) | 1.13 (0.85-1.51) |  |
| Gestational diabetes | *Yes* | 65 (76.5) | 20 (23.5) | 1.16 (0.69-1.94) | 0.58 |
|  | *No* | 1278 (79.0) | 340 (21.0) | 1 |  |
| Type of delivery | *Spontaneous* | 975 (76.6) | 262 (21.2) | 1 | 0.95 |
|  | *Caesarean* | 368 (73.2) | 98 (21.0) | 0.99 (0.76-1.29) |  |
| Newborn gender | *Male* | 685 (80.0) | 171 (20.0) | 0.87 (0.69-1.10) | 0.24 |
|  | *Female* | 658 (77.7) | 189 (22.3) | 1 |  |
| Mother smoker | *Yes* | 121 (77.6) | 35 (22.4) | 1.09 (0.73-1.62) | 0.68 |
|  | *No* | 1222 (79.0) | 325 (21.0) | 1 |  |
| Exclusive breastfeeding ≥ 6 months | *Yes* | 321 (77.0) | 96 (23.0) | 1 | 0.52 |
|  | *No* | 938 (78.5) | 257 (21.5) | 0.92 (0.70-1.20) |  |
| Breastfeeding at 12 months | *Yes* | 598 (78.0) | 137 (18.6) | 1 | 0.02 |
|  | *No* | 726 (73.7) | 219 (23.2) | 1.32 (1.04-1.67) |  |
| Breastfeeding at 24 months | *Yes* | 214 (80.8) | 51 (19.2) | 1 | 0.42 |
|  | *No* | 1123 (78.5) | 307 (21.5) | 1.15 (0.82-1.60) |  |
| Timing of complementary food introduction (months) | *<5* | 192 (76.2 | 60 (23.8) | 1.25 (0.89-1.76) | 0.44 |
|  | *5* | 580 (78.7) | 157 (21.3) | 1.08 (0.84-1.39) |  |
|  | *≥6* | 571 (80.0) | 143 (20.0) | 1 |  |
| Baby-led weaning | *Yes* | 309 (78.6) | 82 (21.0) | 1 | 0.88 |
|  | *No* | 1026 (73.2) | 278 (21.3 | 1.02 (0.77 -1.35) |  |
| Child care attendance | *Yes* | 859 (79.4) | 223 (20.6) | 1 | 0.48 |
|  | *No* | 484 (77.9) | 137 (22.1) | 1.09 (0.86-1.39) |  |
| Time spent outdoors | *Low* | 280 (71.9) | 71 (20.2) | 0.89 (0.63-1.25) | 0.80 |
|  | *Medium* | 652 (76.7) | 178 (21.4) | 0.96 (0.73-1.26) |  |
|  | *High* | 362 (80.3) | 103 (22.2) | 1 |  |
| Infant appetite*** | *Poor* | 101 (90.1) | 5 (4.7) | 0.21 (0.08-0.51) | **<0.001** |
|  | *Normal* | 1142 (75.8) | 274 (19.4) | 1 |  |
|  | *Big* | 78 (44.7) | 76 (49.4) | 4.06 (2.88 -5.72) |  |
| Parents concerned about infant growth | *Yes* | 228 (84.3) | 31 (12.0) | 0.47 (0.31-0.69) | **0.0001** |
|  | *No* | 1065 (75.2) | 310 (22.5) | 1 |  |
| Frequency of video watching | *Low* | 116 (75.9) | 19 (14.1) | 1 | **0.002** |
|  | *Medium* | 655 (80.6) | 158 (19.4) | 1.47 (0.88-2.47) |  |
|  | *High* | 518 (74.7) | 175 (25.3) | 2.06 (1.23-3.45) |  |
| Frequency of direct interaction with devices | *Low* | 322 (82.8) | 69 (17.6) | 1 | 0.10 **(0.03)*** |
|  | *Medium* | 719 (78.3) | 199 (21.7) | 1.29 (0.95-1.75) |  |
|  | *High* | 268 (75.9) | 85 (24.1) | 1.48 (1.04-2.11) |  |
| Sleep disorders | *Yes* | 440 | 129 (22.7) | 1.12 (0.88-1.43) | 0.37 |
|  | *No* | 859 | 225 (20.8) | 1 |  |
| Overweight at 12 months | *Yes* | 195 | 193 (49.7) | 6.80 (5.26-8.80) | **<0.0001** |
|  | *No* | 1148 | 167 (12.7) | 1 |  |

** p-value of chi-square for trend test.* **Educational level: low: no schooling or primary versus high: secondary school or university. ***Infant appetite as perceived by the parents.

**Supplementary Table 3 - Association between maternal and neonatal characteristics and persistent overweight**

| **Variable** | ***Value*** | **Persistent overweight** | | **OR (95%CI)** | **p-value** |
| --- | --- | --- | --- | --- | --- |
|  |  | No  (N=1,551) | Yes  (N=152) |  |  |
| Geographical area of residence | *North* | 777 (92.0) | 68 (8.0) | 1 | **0.02** |
|  | *Center* | 294 (93.3) | 21 (6.7) | 0.82 (0.49-1.36) |  |
|  | *South* | 480 (88.4) | 63 (11.6) | 1.50 (1.05-2.15) |  |
| Setting | *Urban* | 571 (81.2) | 55 (8.8) | 1 | 0.88 |
|  | *Rural* | 980 (81.0) | 97 (9.0) | 1.03 (0.73-1.45) |  |
| Maternal age at delivery | *Median (Interquartile range)* | 33.0  (30.0-36.5) | 32.0  (29.0-36.0) | Not applicable | 0.38 |
|  | *Mean (SD)* | 33.1 (5.4) | 32.7 (5.2) |  |  |
| Educational level* | *High* | 1322 (91.3) | 126 (8.7) | 1 | 0.51 |
|  | *Low* | 216 (90.0) | 24 (10.0) | 1.17 (0.74-1.85) |  |
| Employment status | *Employed* | 1116 (91.9) | 98 (8.1) | 1 | 0.05 |
|  | *Unemployed* | 427 (89) | 53 (11.0) | 1.41 (0.99-2.01) |  |
| Mother born in Italy | *Yes* | 1373 (91.4) | 130 (8.6) | 1 | 0.36 |
|  | *No* | 177 (89.4) | 21 (10.6) | 1.25 (0.77-2.04) |  |
| Marital status | *With partner* | 1541 (91.1) | 150 (8.9) | 1 | 0.29 |
|  | *Single* | 10 (83.3) | 2 (16.7) | 2.05 (0.45-9.46) |  |
| Primiparous | *Yes* | 818 (91.7) | 74 (8.3) | 0.86 (0.62-1.21 | 0.39 |
|  | *No* | 725 (90.5) | 76 (9.5) | 1 |  |
| Pre-pregnancy BMI | *Underweight* | 106 (97.2) | 3 (2.8) | 0.30 (0.09-0.96) | **0.01** |
|  | *Normal* | 1048 (91.4) | 99 (8.6) | 1 |  |
|  | *Overweight* | 364 (88.3) | 48 (11.7) | 1.40 (0.97-2.01) |  |
| Gestational weight gain | *Below* | 524 (91.4) | 49 (8.6) | 1.00 (0.67-1.49) | 0.57 |
|  | *Normal* | 598 (91.4) | 56 (8.66) | 1 |  |
|  | *Over* | 386 (89.8) | 44 (10.2) | 1.22 (0.80-1.84) |  |
| Gestational diabetes | *Yes* | 79 (92.9) | 6 (7.1) | 0.77 (0.33-1.79) | 0.54 |
|  | *No* | 1472 (91.0) | 146 (9.0) | 1 |  |
| Type of delivery | *Spontaneous* | 1131 (76.6) | 106 (8.6) | 1 | 0.40 |
|  | *Caesarean* | 420 (73.2) | 46 (9.9) | 1.17 (0.81-1.68) |  |
| Newborn gender | *Male* | 785 (91.7) | 71 (8.3) | 0.86 (0.61-1.19) | 0.36 |
|  | *Female* | 766 (90.4) | 81 (9.6) | 1 |  |
| Mother smoker | *Yes* | 139 (89.1) | 17 (10.9) | 1.28 (0.75-2.18) | 0.36 |
|  | *No* | 1412 (91.3) | 135 (8.7) | 1 |  |
| Exclusive breastfeeding ≥ 6 months | *Yes* | 373 (89.4) | 44 (10.6) | 1 | 0.26 |
|  | *No* | 1091 (91.3) | 104 (8.7) | 0.81 (0.56-1.17) |  |
| Breastfeeding at 12 months | *Yes* | 676 (92.0) | 59 (8.0) | 1 | 0.20 |
|  | *No* | 852 (90.2) | 93 (9.8) | 1.25 (0.89-1.76) |  |
| Breastfeeding at 24 months | *Yes* | 214 (80.8) | 51 (19.2) | 1 | 0.42 |
|  | *No* | 1123 (78.5) | 307 (21.5) | 1.15 (0.82-1.60) |  |
| Timing of complementary food introduction (months) | *<5* | 227 (90.1) | 25 (9.9) | 1.06 (0.66-1.72) | 0.59 |
|  | *5* | 677 (91.9) | 60 (8.1) | 0.86 (0.59-1.23) |  |
|  | *≥6* | 647 (90.6) | 67 (9.4) | 1 |  |
| Baby-led weaning | *Yes* | 367 (93.9) | 24 (6.1) | 1 | **0.026** |
|  | *No* | 1176 (90.2) | 128 (9.8) | 1.66 (1.06 -1.21) |  |
| Child care attendance | *Yes* | 989 (91.4) | 936 (8.6) | 1 | 0.53 |
|  | *No* | 562 (90.5) | 59 (9.5) | 1.12 (0.79-1.57) |  |
| Time spent outdoors | *Low* | 317 (71.9) | 34 (9.7) | 1.32 (0.80-2.16) | 0.49 |
|  | *Medium* | 754 (76.7) | 76 (9.2) | 1.24 (0.82-1.88) |  |
|  | *High* | 430 (80.3) | 35 (7.5) | 1 |  |
| Infant appetite** | *Poor* | 105 (99.1) | 1 (0.9) | 0.11 (0.02-0.83) | **<0.001** |
|  | *Normal* | 1307 (92.3) | 109 (7.7) | 1 |  |
|  | *Big* | 115 (74.7) | 39 (25.3) | 4.07 (2.69 -6.14) |  |
| Parents concerned about infant growth | *Yes* | 243 (84.3) | 16 (6.2) | 0.67 (0.39-1.15) | 0.14 |
|  | *No* | 1252 (75.2) | 123 (8.9) | 1 |  |
| Frequency of video watching | *low* | 128 (94.8) | 7 (5.2) | 1 | 0.17 |
|  | *medium* | 743 (91.4) | 70 (8.6) | 1.72 (0.77-3.83) |  |
|  | *high* | 623 (89.9) | 70 (10.1) | 2.05 (0.92-4.57) |  |
| Frequency of direct interaction with devices | *low* | 372 (82.8) | 19 (4.9) | 1 | **0.006** |
|  | *medium* | 825 (78.3) | 93 (10.1) | 2.21 (1.33-3.67) |  |
|  | *high* | 317 (75.9) | 36 (10.2) | 2.22 (1.25-3.95) |  |
| Sleep disorders | *Yes* | 520 (91.4) | 49 (8.6) | 0.92 (0.64-1.31) | 0.64 |
|  | *No* | 983 (90.7) | 101 (9.3) | 1 |  |

*** Educational level: low: no schooling or primary versus high: secondary school or university. **Infant appetite as perceived by the parents.
